# MRI as a tool to assess surgical margins and pseudocapsule features directly following partial nephrectomy for small renal masses

**DOI:** 10.1007/s00330-018-5630-9

**Published:** 2018-07-24

**Authors:** Tim J. van Oostenbrugge, Willemien Runneboom, Elise Bekers, Jan Heidkamp, Johan F. Langenhuijsen, Andor Veltien, Arie Maat, Peter F. A. Mulders, Christina A. Hulsbergen-van de Kaa, Jurgen J. Fütterer

**Affiliations:** 1 Department of Urology, Radboud University Medical Center, P.O. Box 9101, 6525 GA Nijmegen, The Netherlands; 2 Department of Radiology and Nuclear Medicine, Radboud University Medical Center, Nijmegen, The Netherlands; 3 Department of Pathology, Radboud University Medical Center, Nijmegen, The Netherlands

**Keywords:** Kidney neoplasms,, Renal cell carcinoma,, Magnetic resonance imaging,, Margins of excision

## Abstract

**Purpose:**

To evaluate the feasibility of ex vivo 7T MRI to assess surgical margins (SMs) and pseudocapsule (PC) features after partial nephrectomy (PN).

**Materials and methods:**

In this prospective, IRB-approved study, seven patients undergoing a PN for nine tumours between November 2014 and July 2015 were included for analysis after obtaining informed consent. MRI of the specimen was acquired using a 7T small bore scanner. The imaging protocol consisted of anatomical T1-, T2- and diffusion-weighted imaging. After formalin fixation, specimens were cut for pathology work-up in the same orientation as the MR images were obtained. The entire specimen was processed into H&E slides that were digitally scanned, annotated and correlated with radiological findings for negative SMs, PC presence, PC continuity and extra-PC-extension (EPCE). Sensitivity and specificity of MRI for assessment of these endpoints were calculated.

**Results:**

The sensitivity and specificity for assessment of the SM were 100% and 75%, respectively. Two false-positive outcomes were reported, both in case of EPCE and a SM ≤0.5 mm. For the presence of a PC, sensitivity and specificity were 100% and 33%, respectively. Two false-positive scans with anatomical structures mimicking the presence of a PC occurred. If a PC was present, continuity and EPCE were assessed with a sensitivity and specificity of 75% and 100% and 67% and 100%, respectively.

**Conclusion:**

Ex vivo 7T MRI is a feasible tool for perioperative evaluation of SMs, and if present, PC features after PN. This may facilitate maximal sparing of renal parenchyma without compromising oncological outcomes.

**Key Points:**

*• Ex vivo MRI may contribute to improvement of negative surgical margins during partial nephrectomy.*

*• Due to the assessment of surgical margins within a limited time span from obtaining the partial nephrectomy specimen, surgery for more complex tumours is possible with maximum sparing of healthy renal parenchyma without compromising oncological outcomes.*

*• The intra operative assessment of pseudocapsule continuity along the resection margin enables maximal sparing of healthy renal parenchyma without delayed diagnosis of incomplete resection.*

## Introduction

The standard of care for renal masses not exceeding 7 cm in diameter is partial nephrectomy (PN) whenever feasible [1]. A negative surgical margin (SM) is one of the key trifecta outcomes during PN, next to minimal renal function decrease and the absence of complications [2]. This challenges the urologist to minimise SMs in order to maximally preserve healthy renal parenchyma without compromising oncological outcomes [3].

With the narrowing of SMs the need for intraoperative SM assessment grows. This includes an accurate assessment of the tumour pseudocapsule (PC) present in the majority of malignant renal tumours [4], mainly because enucleation along the plane between the tumour PC and the renal parenchyma is an established approach to preserve healthy parenchyma [3]. Despite its relevance, intra-operative SM and PC assessment remains a challenging task. In particular, because frozen section analysis is considered not to be contributory due to several studies evaluating 380 cases that failed to show any true positive results [5–7]. Therefore, to assess SMs, the use of ex vivo specimen imaging might be of added value. We hypothesized that ex vivo MRI of renal tumour specimens is a feasible tool to assess SMs and PC features following PN.

The objective of this study was to evaluate the feasibility of ex vivo 7T MRI to assess SMs and PC features after PN and to correlate our findings with whole mount section histopathology as the gold standard.

## Materials and methods

### Recruitment

The Institutional Review Board approved this study. All patients gave written informed consent. Data were prospectively collected between November 2014 and July 2015. Inclusion criteria were a clinically suspicious tumour for RCC, scheduled PN and obtained written informed consent. No exclusion criteria were imposed. Patients were recruited and consecutively selected at the urology outpatient clinic. In total, ten specimens containing 11 tumours from nine patients were included. One specimen contained an incidentally detected second tumour that was included for analysis. One specimen consisted of a benign cyst and in one specimen perioperative disintegration of the resection margin occurred, both were excluded from analysis (Table 1).

**Table 1:** Summarized findings (where applicable findings reported reflect radiological/pathological findings)

Specimen	Resection technique	Subtype	WHO/ISUP nuclear grading	Maximal tumour diameter (mm)	Surgical margin	Smallest resection margin (mm)	Pseudocapsule present	Continuity pseudocapsule	Extra pseudocapsulair extension	Follow-up (months)^4^	Local recurrence
I	Resection	Oncocytoma	n.a.	20/20	Neg/Neg	9.0/1.5	Yes/No	Yes/n.a.	No/n.a.	0	No
II	Enucleoresection	Oncocytoma	n.a.	21/15	Neg/Neg	2.3/1.2	No/No	n.a./n.a.	n.a./n.a.	34^5^	No
III	Resection	Clear cell	II	36/50	Pos/Pos	n.a./n.a.	Yes/Yes	No/No	No/No	33	No
IV^1^	Resection	Chromophobe	n.a.	x/x	x/x	x/x	x/x	x/x	x/x	27	No
V	Pure enucleation	Clear cell	II	9.7/5.0	Neg/Neg	3.7/0.5	Yes/Yes	Yes/Yes	No/No	4	No
VI	Resection	Clear cell	III	21/20	Neg/Neg	0.2/0.6	Yes/Yes	Yes/No (70% intact)	No/Yes	4	No
VII^2^	Resection	Benign cyst	n.a.	x/x	x/x	x/x	x/x	x/x	x/x	21	No
VIII	Hybrid enucleation	Papillary type I	II	59/70	Neg/Neg	3.0/ 0.3	Yes/No	Yes/n.a.	No/n.a.	23	No
IX	Resection	Clear cell	II	31/30	Pos/Neg	n.a./0.5.	Yes/Yes	No/No	Yes/Yes	25	No
X	Pure enucleoresection	Clear cell	II	23/24	Neg/Neg	1.6/0.4	Yes/Yes	Yes/Yes	No/No	25	No
X^3^	Resection	Papillary type I	II	11/10	Pos/Neg	n.a./0.5.	Yes/Yes	No/No	Yes/Yes	25	No

1. Fragmented specimen with disintegration of the resection margin, therefore surgical margins and pseudocapsule could not be assessed on MRI and histopathology

2. Only a small benign cyst was visualized on ex vivo MRI, pathology report confirmed this finding. Patient was excluded from further analysis

3. Incidentally detected second tumour in specimen X

4. Measured from the date of surgery until the last moment of follow-up imaging of the affected kidney

5. Follow-up performed for second incidentaloma detected after surgery

*WHO/ISUP* World Health Organization/International Society of Urological Pathology

### Surgery

PN was performed by open or robotic approach. Before resection, warm ischaemia time was applied by clamping the renal artery. Tumour resection was done with a minimal healthy tissue margin technique. If feasible, enucleation along the tumour PC was performed without resecting healthy parenchyma. The resection technique was macroscopically scored by one observer (TO) as pure enucleation, hybrid enucleation, pure enucleoresection or hybrid enucleoresection or resection according to the surface–intermediate–base margin score [8].

### Specimen MR imaging

**Fig. 1:**
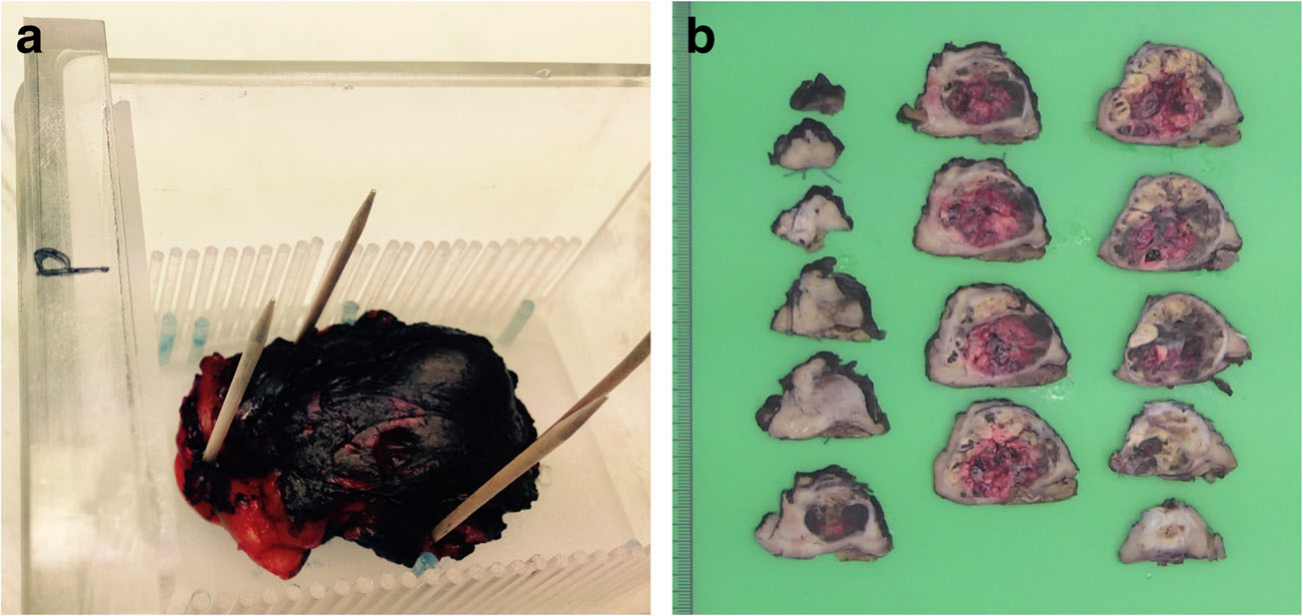
(**a**) Specimen I containing a 20-mm large oncocytoma, fixated on a block of paraffin inside a customised Perspex holder with a Perspex row of pins on both sides 3 mm apart, used for pathology slicing after fixation in formalin. The holder also contains seven (two posterior left) water-filled tubes (blue pins) to facilitate matching between MR imaging and histopathology slides. The total setup is positioned in a glass container. (**b**) After MR examination the oil in the glass container was disposed of and the specimen was fixated at least 24 h in formalin. Subsequently, the specimen was cut in 3- to 5-mm thick whole mount sections from anterior to posterior using the pins in the holder and totally included for pathology work-up

Following resection, the specimen was transported to the MR suite. After inking the parenchymal SM and renal capsule site, the specimen was fixated with wooden pins on a paraffin block within a customised Perspex holder containing rows of Perspex pins 3 mm apart from each other (Fig. 1). This holder and pins were designed to align imaging orientation with pathology slicing. The complete setup was positioned in a glass container. The specimen was immersed in perfluropolyether oil (Galden, Solvay Solexis) to avoid susceptibility artefacts. MR imaging was acquired using a 7T horizontal wide bore (200 mm) ClinScan animal scanner interfaced to a clinical Siemens Syngo VB15 console (Bruker BioSpin). MR images were recorded using an integrated circular polarized transmit/receive 1H volume coil with a free inner diameter of 154 mm. To match the MR and histopathology slides, MR sequences were aligned with water-filled tubes in the Perspex holder. The imaging protocol contained anatomical T1-weighted 3D gradient echo, T2-weighted turbo spin-echo (TSE) sequences and diffusion-weighted imaging (DWI) with b-values of 0–100–500–1,000 s/mm^2^ (see Table 2 for imaging parameters). Apparent diffusion coefficient (ADC) maps were calculated using the standard ADC post-processing available on the console. The number of slices was chosen to entirely cover the specimen. The total work-up time, measured from the time of the specimen removal until availability of the images, was recorded.

**Table 2:** MR imaging parameters

Sequence	Slides	TR (ms)	TE (ms)	FA (degrees)	ST (mm)	Averages	matrix	FOV (mm)	Pixel size (mm)	Scan time (min:s)
TSE	25-77	3,842–13,940	27	180	1	1	256*256	50*50	0.19*0.19	2:26–7:30
3D GRE	104-256	15	2.8	15	0.3	1	128–320*128–320	49–80*49–80	0.26*0.26	3:39–8:58
DWI	25-88	2,000	48	180	1	4	128*128	50*50	0.39*0.39	13:20–46:56

*TSE* turbo spin-echo, *GRE* gradient echo, *DWI* diffusion-weighted imaging, *TR* repetition time, *TE* echo time, *FA* flip angle, *ST* slice thickness, *FOV* field of view

Variations in TR, number of slices and scan time were caused by variations in volume of the specimens. With changing specimen volumes, matrix and FOV were adjusted for the third GRE sequence to maintain the voxel size

### MR imaging report

MR images were reviewed by a radiologist (JF) blinded for the pathology outcome with 15 years of experience in the field of MR imaging. Tumour size, smallest SM (defined as smallest distance from the tumour border towards parenchymal SM), SM negativity, presence or absence of a PC, and if present, continuity of the PC and extra PC extension (EPCE) were evaluated (Table 1).

### Image quality assessment

To establish which sequence was most suitable for evaluation, the quality of the MR images was assessed by the reviewing radiologist (JF), who evaluated the specimen characteristics. Visual quality assessment was done by scoring visibility of SM and PC features using a 5-point scale for all used sequences. Score 1 reflected excellent visualisation; score 5 reflected the images as being non-diagnostic.

### Pathology workflow

After the MR imaging the specimen maintained its position on the board. After at least 24 h of fixation in formalin it was cut in the same direction the MR images were obtained. To enable proper correlation between the MR images and histopathological slides, the landmarks on the board were used. The specimen slides obtained were paraffin embedded after which 4 μm haematoxylin and eosin slides were prepared. All tumour-containing slides were digitally scanned (3DHistech Panoramic250, SYSMEX Belgium N.V., and Olympus Dotslide, Olympus Nederland B.V.). While being blinded for the MR imaging results, histopathological annotation on all scanned slides was done by two residents in pathology (WR/EB) under supervision of a dedicated uropathologist with 25 years of experience (CH) using commercially available software (Aperio ImageScope v11.2.0.780, Aperio Technologies, Inc. and OlyVIA, Olympus).

### Pathology report

All features as reported by the radiologist were also histologically evaluated. In addition, histological RCC subtype according to the 2016 World Health Organization (WHO) classification and International Society of Urological Pathology (ISUP) nuclear grading were reported [9, 10]. On histopathology a PC was defined as a band of fibrous connective tissue located at the interface of the tumour and adjacent renal parenchyma [11] (Table 1). EPCE was defined as complete penetration of the tumour through the PC. The following conditions were not considered EPCE: intact PC with tumour invasion, incomplete PC but no tumour protrusion in the renal parenchyma, and PC with protruding but no penetrating tumour [12]. A positive SM was defined as presence of tumour cells in the inked surface of the parenchymal resection border.

### Statistical analysis

For descriptive analysis median (range) values were used. Correlation between the radiological and pathological review for tumour diameter and smallest SM was established using Pearson’s r. A *p*-value <0.05 was considered statistically significant. Sensitivity and specificity of MRI were calculated for assessment of PC and SM features.

## Results

In total, nine tumours in eight specimens obtained from seven patients were available for analysis. Five (71%) patients were male, median age was 65 years (range 56–73). Robot-assisted partial nephrectomy (RAPN) and open surgery were performed in six and one patients, respectively. Median tumour diameter on radiological and pathological review were 21 mm (range 10–59) and 20 mm (5–70), respectively (Pearson’s r 0.978; *p* < 0.01). The total work-up time, measured from the time of the specimen removal until availability of the relevant images, was 80 min (range 42–186).

### Visual quality assessment

**Fig. 2:**
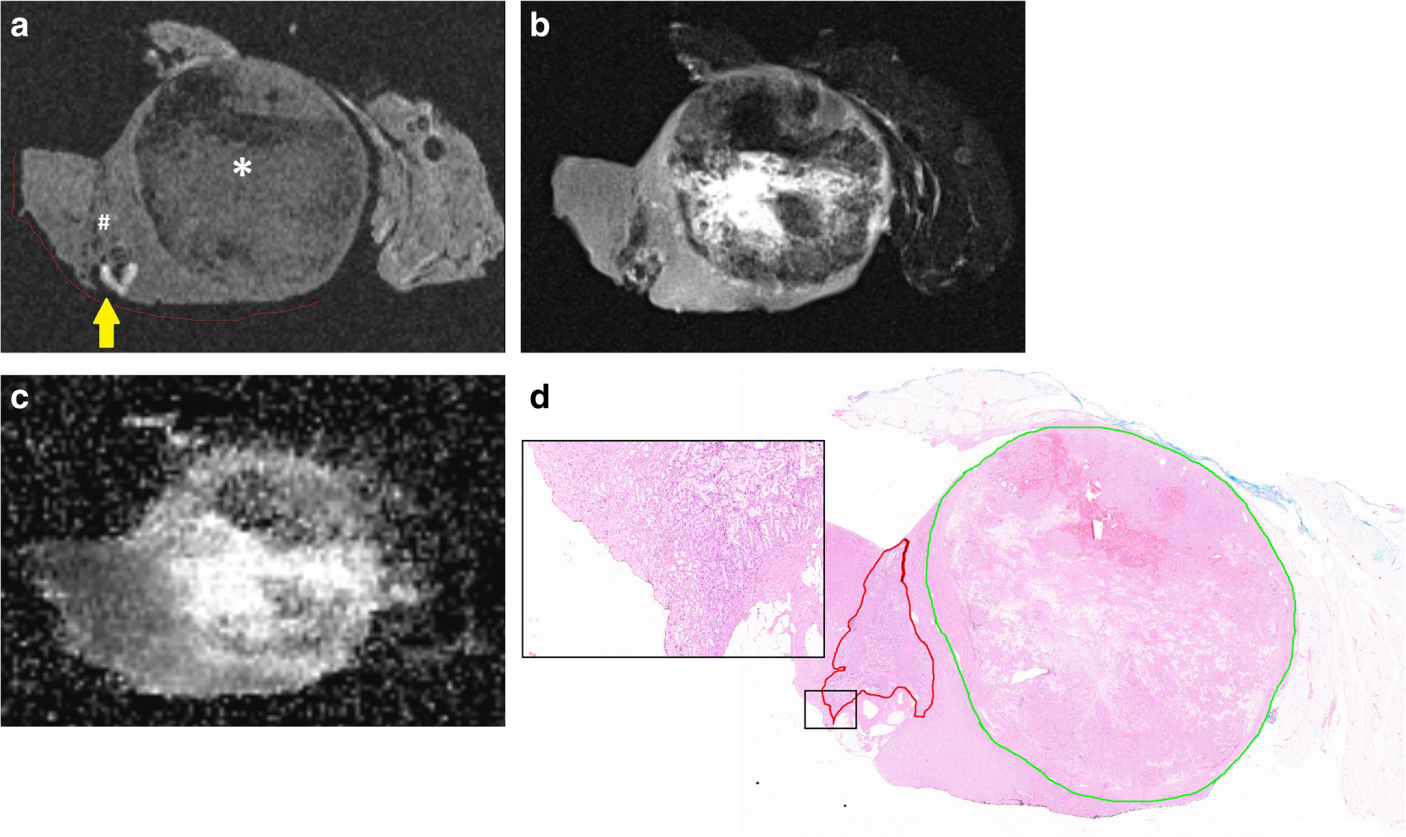
(**a**) Specimen X containing a 24-mm large clear cell tumour and a 10-mm large incidentally detected papillary tumour. The T1-weighted images show the clear cell (*) and papillary tumour (#), the surgical margin (red line), and suspected positive surgical margin (yellow arrow.) Image quality was scored as ‘3 – acceptable’. (**b**) According to T2-weighted images. Image quality was scored as ‘1 – excellent’. (**c**) According to calculated ADC map. Image quality was scored as ‘4 – poor’. (**d**) Histopathological slide with enlargement shows demarcation of the clear cell (green) and papillary (red) tumour. The enlargement does not confirm tumour cells in the resection border

Median scores for ability to assess SM and PC features were excellent (score 1) using T2-weighted images. T1-weighted images median scores were acceptable (score 3) with outliers to non-diagnostic for assessment of both features. DW images were least usable with median scores being acceptable (score 3) and poor (score 4) for assessment of SM and PC features, respectively (Fig. 2 and Table 3).

**Table 3:** Visual quality assessment of used sequences to assess surgical margins (SMs) and pseudocapsule (PC) features

	Median score	Range
T1-weighted for SM	Acceptable	Acceptable – excellent
T1-weighted for PC	Acceptable	Acceptable
T2-weighted for SM	Excellent	Excellent
T2-weighted for PC	Excellent	Excellent
DWI for SM	Acceptable	Non-diagnostic – acceptable
DWI for PC	Poor	Poor

Possible outcomes: non-diagnostic, poor, acceptable, good, excellent

*SM* surgical margins, *PC* pseudocapsule, *DWI* diffusion-weighted imaging

### Surgical margins

**Fig. 3:**
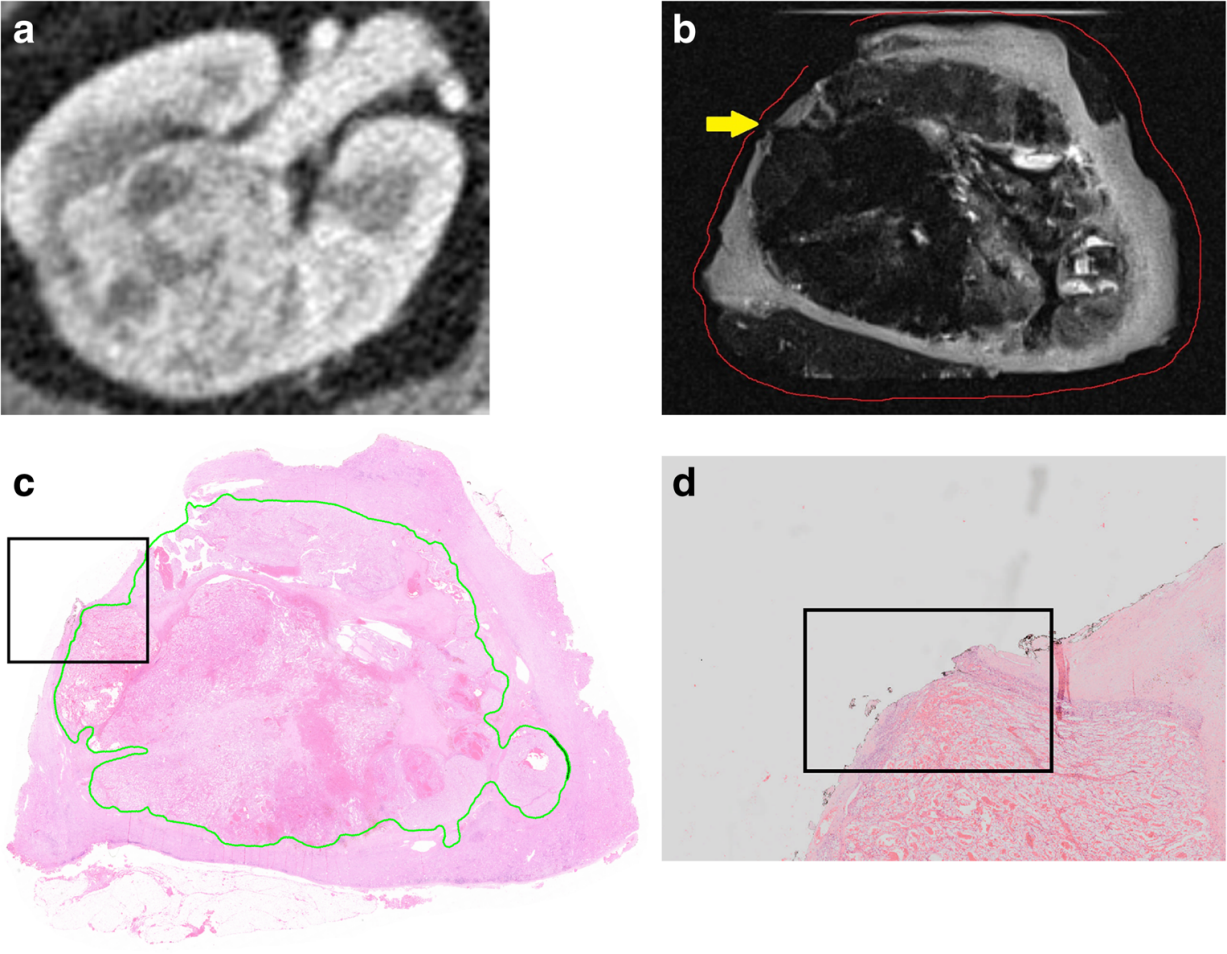
(**a**) Preoperative CT scan of specimen III showing a 50-mm large clear cell RCC in the right kidney. (**b**) The T2-weighted scan of the specimen after resection showed suspicion for a positive (yellow arrow) surgical margin (red line). (**c**) Histopathological slide confirmed the 1.4-mm large positive surgical margin. Black box is enlarged in Fig. 3d. (**d**) The 1.4-mm large positive surgical margin in detail

Median smallest SM was 2.7 mm (0.2–9.0) and 0.6 mm (0.3–1.5) on radiological and pathological review (Pearson’s r 0.667; *p* = 0.147). One SM was on histological evaluation found to be positive, which was identified as such on MR imaging (Fig. 3). Two tumours showed a false-positive SM on MR imaging. Presence of tumour cells in the SM was not confirmed on histopathology in one case with a smallest SM of 0.5 mm (Fig. 2). One specimen showed EPCE and ingrowth of the tumour in a blood vessel towards the parenchymal resection border. Additional histopathological slides were cut but the SM remained negative with the smallest margin being 0.5 mm. The sensitivity and specificity for assessment of negative SM with MR imaging were 100% (9/9) and 75% (6/8), respectively.

### Pseudocapsule

**Fig. 4:**
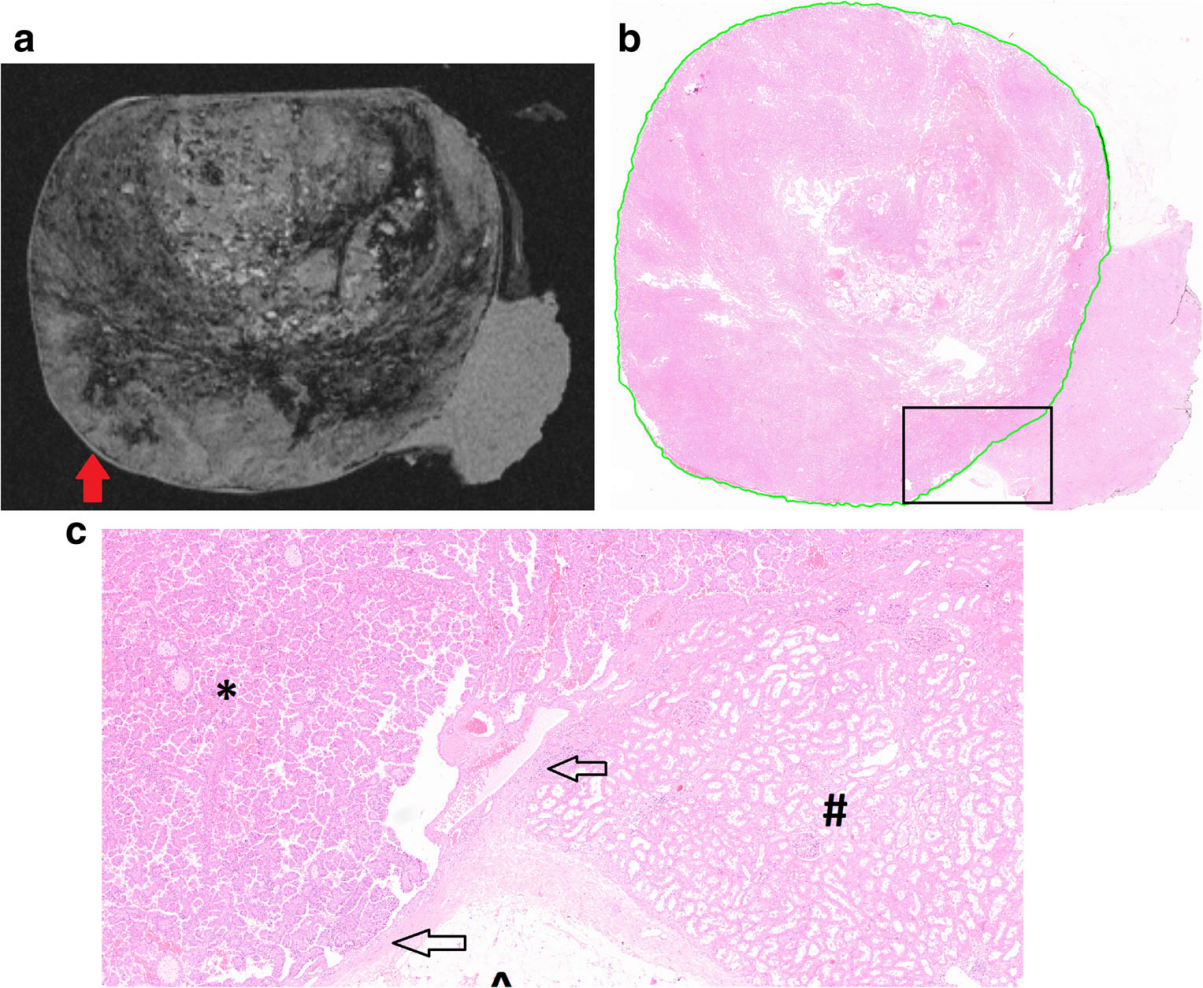
(**a**) Specimen VIII containing a 70-mm papillary tumour showing a false-positive result for presence of a pseudocapsule (red arrow) on the T1-weighted scan; the specimen is slightly compressed at the top to fit in the setup. (**b**) The annotated histopathological slide shows the tumour borders (green line). The black square is enlarged in **c**. (**c**) The structure marked as a pseudocapsule on MRI was found to be a thin epithelial layer surrounding the tumour mimicking a pseudocapsule. Markers are as follows: * tumour tissue; # renal parenchyma, ^ adipose tissue; arrows mark the epithelial layer

A PC was histologically confirmed in six out of nine cases. Presence or absence of a PC was correctly identified on MRI in seven out of nine cases (78%); two scans were false positive. In both cases an anatomical structure mimicking a tumour PC was present. The first case concerned a papillary RCC outlined by a thin epithelial layer of a cystic space, thereby creating a delicate space that mimicked a PC (Fig. 4). In the second specimen, the renal capsule at the perirenal site together with longitudinal cut blood vessels and compressed parenchyma towards the surgical margin mimicked a PC. Sensitivity and specificity for presence of a PC were 100% (6/6) and 33% (1/3), respectively.

Histopathologically determined PCs were discontinuous in 67% (4/6) and EPCE was present in 50% (3/6), respectively. In one case both PC discontinuity and EPCE were not identified as such on MRI. Sensitivity and specificity for continuity and EPCE were 75% (3/4) and 100% (2/2) and 67% (2/3) and 100% (3/3), respectively.

## Discussion

We showed that MRI is a feasible and sensitive tool to assess SMs following partial nephrectomy. Detection of PC presence is challenging due to anatomical structures mimicking its appearance. If a PC is present, accurate evaluation of continuity and EPCE is possible with a sensitivity of 75% and 67% and specificity of 100% for both features. T2-weighted images provide the best quality for evaluation.

Intraoperative evaluation of SMs plays an important role in the emerging field of PN. Positive surgical margins are reported in 0–7% after PN [13]. Enucleation can be used as a resection technique to facilitate maximal parenchymal sparing in order to preserve renal function. However, it harbours the risk of incomplete resection mostly due to PC absence or discontinuity in several tumour subtypes [4, 11–13]. Also, PN performed in an imperative indication, i.e. large tumours and tumours with an unfavourable localisation, are prone to non-radical resection, with 18 % positive SMs reported in the literature [13, 14]. Especially in these cases, ex vivo imaging to assess the SM can facilitate the surgeon in sparing healthy renal parenchyma without compromising oncological outcomes.

Previously, the feasibility for SM assessment using ex vivo ultrasound (US) has been evaluated. In three studies concerning 118 cases a specificity of 100% was found. The high sensitivity in these studies (97–100%) should be considered with caution because each study only contained one positive SM [15–17]. The disadvantage of US is the user dependency, making it prone to interobserver variability. This makes these studies difficult to reproduce. Also, compression of the specimen can cause tissue deformations on imaging leading to misinterpretation of SMs. In case of absence of PC, US imaging can be challenging because tumour echogenicity may be similar to that of parenchyma, while MRI provides better soft tissue contrast. Moreover, US imaging is hard to accurately correlate with final histopathology because the imaging plane cannot be correlated with the direction used for pathology slicing.

A more recent development is ex vivo fluorescence imaging of the renal specimen after PN. The use of indocyanine green in 16 patients showed a sensitivity and specificity of 100% for both for SM assessment. However, this cohort contained no positive SMs [18]. Another recent study confirmed the feasibility of ex vivo fluorescence imaging using IRDye800CW in six patients undergoing PN in whom one positive SM was correctly identified [19]. An advantage of fluorescence imaging is that imaging before, during and after tumour resection can easily be combined [20].

The work-up time measured in this study was 80 min (range 42–186). A large part of this time span involved the time that was needed to optimise the scanning protocol and to assure rigid methods to test the technical feasibility. If implemented in clinical use, mounting of the specimen should take about 2 min when done on site. This is in contrast to our study, where mounting of the specimen was done at the pathology laboratory to assure correct correlation with final histopathology. Subsequent positioning in the MR scanner, adjustment and reference imaging should take about 2–3 min. We suggest using the T2-weighted sequence for which the scanning time is 3–7 min, depending on the specimen size. Reviewing the MR images should also take about 2 min. The rest of the total work-up time is dependent on logistical factors such as location of the MRI and availability of a radiologist to review the MR images. A total work-up should be feasible within 10–15 min when optimal conditions are created.

A limitation to this study is the small sample size. Reported sensitivity should be interpreted cautiously because only one case with a positive SM was included. The specificity of 75% for SM assessment was hampered due to two cases showing a false-positive result. Both cases had EPCE and a very small resection margin (≤0.5 mm). However, the detection of a 1.4-mm large positive SM indicates that larger positive SMs, which are more important in terms of prognosis, can be detected using MR imaging [13]. Future studies with larger sample sizes that will test the feasibility of this technique on the commonly available 3T MRI should be conducted.

Ex vivo 7T MRI is a feasible tool for perioperative evaluation of SMs, and if present PC features after PN. This may facilitate maximal sparing of renal parenchyma without compromising oncological outcomes.

## Abbreviations

7T: 7 Tesla
ADC: Apparent diffusion coefficient
DWI: Diffusion-weighted imaging
EPCE: Extra-pseudocapsular-extension
H&E: Haematoxylin and eosin
IRB: Institutional Review Board
ISUP: International Society of Urological Pathology
MRI: Magnetic resonance imaging
PC: Pseudocapsule
PN: Partial nephrectomy
RCC: Renal cell carcinoma
SM: Surgical margin
TSE: Turbo spin-echo
US: Ultrasound
WHO: World Health Organization
